# Acoustic Emission and Echo Signal Compensation Techniques Applied to an Ultrasonic Logging-While-Drilling Caliper

**DOI:** 10.3390/s17061351

**Published:** 2017-06-10

**Authors:** Yongchao Yao, Xiaodong Ju, Junqiang Lu, Baiyong Men

**Affiliations:** State Key Laboratory of Petroleum Resources and Prospecting, China University of Petroleum, Beijing 102249, China; juxdong@cup.edu.cn (X.J.); lujq@cup.edu.cn (J.L.); bymen@cup.edu.cn (B.M.)

**Keywords:** logging-while-drilling caliper, ultrasonic distance measurement, piezoelectric transducer, blind zone elimination, time-varying amplification

## Abstract

A logging-while-drilling (LWD) caliper is a tool used for the real-time measurement of a borehole diameter in oil drilling engineering. This study introduces the mechanical structure and working principle of a new LWD caliper based on ultrasonic distance measurement (UDM). The detection range is a major performance index of a UDM system. This index is determined by the blind zone length and remote reflecting interface detection capability of the system. To reduce the blind zone length and detect near the reflecting interface, a full bridge acoustic emission technique based on bootstrap gate driver (BGD) and metal-oxide-semiconductor field effect transistor (MOSFET) is designed by analyzing the working principle and impedance characteristics of a given piezoelectric transducer. To detect the remote reflecting interface and reduce the dynamic range of the received echo signals, the relationships between the echo amplitude and propagation distance of ultrasonic waves are determined. A signal compensation technique based on time-varying amplification theory, which can automatically change the gain according to the echo arrival time is designed. Lastly, the aforementioned techniques and corresponding circuits are experimentally verified. Results show that the blind zone length in the UDM system of the LWD caliper is significantly reduced and the capability to detect the remote reflecting interface is considerably improved.

## 1. Introduction

Logging-while-drilling (LWD) is a well logging technology used in drilling wells. The rock physical parameters and petroleum engineering parameters (e.g., lithology, porosity, permeability, saturation, borehole diameter) of underground formations can be measured in real time by using this technology [1,2,3]. At present, most loggings in highly-deviated wells, horizontal wells, and offshore wells rely on LWD.

Unlike in conventional cable logging, the LWD tools are mounted directly on drill collars [4]. While drilling wells, LWD tools are brought into the target layers to measure specific parameters. Drill collars rotate at a high speed during well drilling, and traditional calipers with mechanical eccentering arms can barely fulfill the measurement tasks. By contrast, a borehole diameter measurement system based on ultrasonic distance measurement (UDM) has many advantages, including non-contact, high speed, and simple information processing [5,6]. Hence, such system is more suitable for borehole diameter measurement during well drilling. Data obtained from caliper logs play an important role in lithology discrimination, borehole correction and cement consumption estimation.

The existence of a measurement blind zone is a major technical defect in a UMD system, which causes several difficulties in measuring near the reflection interface. The blind zone length in a LWD caliper tool that was recently developed by Sinopec Corp is 25 mm [7], and this performance index in most other similar tools is no better. In recent years, slim hole (diameters less than 220 mm) logging and drilling are becoming more and more popular, and a large blind zone is harmful to the measurement near the reflection interface. Thus, a further reduction of the blind zone is necessary. Normally, the length of the blind zone is determined by the impedance characteristics of the acoustic transducer and the performance of the acoustic emission circuit. Therefore, the emission circuit must be designed appropriately to reduce the blind zone length when using a given transducer.

With long travel distance, high mud density, and a low reflection coefficient, ultrasonic attenuation is considerable, and the dynamic range of the echo signal is wide. LWD tools typically have to work in a highly-complicated environment (e.g., strong vibration, high temperature, and high pressure), and tools that are not equipped with mechanical eccentralizers may be affected by adverse situations while working in a borehole, such as serious eccentricity caused by sidewall contact and detection range alteration caused by borehole collapse. To address such situations, the measurement range of the UDM system must be improved, thereby indicating that an ultrasonic caliper should be capable of simultaneously realizing a “zero distance” measurement when the tool comes in contact with the sidewall and the remote reflecting interface measurement when the borehole collapses. 

The rest of the paper is organized as follows: Section 2 introduces the tool structure and basic measurement principles of the ultrasonic LWD caliper. Section 3 discusses the generation mechanism of the blind zone in a UDM system, and studies an acoustic emission technique for reducing the blind zone. Section 4 discusses a signal compensation technique for detecting echo signals that vary in a wide dynamic range, and verifies the technique with several comparative experiments. Finally, some conclusions are put forward in Section 5.

## 2. Tool Structure and Measurement Principle of Ultrasonic LWD Caliper

**Figure 1:**
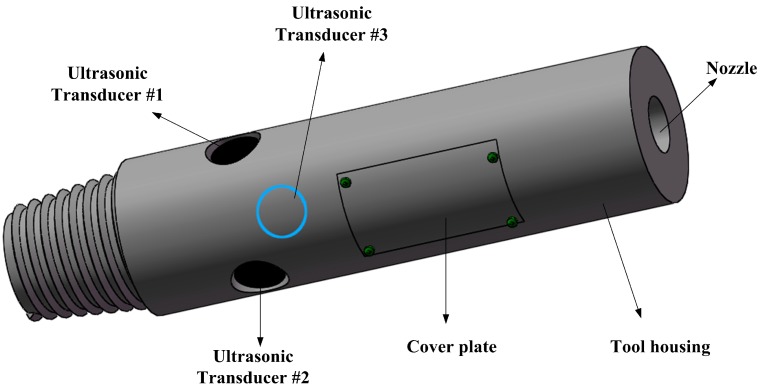
Mechanical profile of the ultrasonic LWD caliper.

Figure 1 illustrates the mechanical profile of the ultrasonic LWD caliper. The outside diameter ranges from 6 to 10 inches (150 mm to 250 mm). On the cross-section of the tool, three cylindrical piezoelectric transducers (Ф 35 mm × 30 mm, 220 kHz) are uniformly embedded into the tool housing at an angle of 120 degrees. The circuit system is installed into the groove on the tool housing and separated from the drilling fluid (borehole mud) by cover plates. The nozzle provides a transport channel for the drilling fluid. 

The transducers transmit ultrasonic waves and receive echoes through the acoustic windows on the tool housing. To reduce the influence on the mechanical strength of the drill collar, and to save space, the number of transducers should be kept to a minimum while satisfying the necessary conditions for borehole diameter measurement. Accordingly, the transducers embedded into the tool housing have to work in the pulse-echo mode. Compared with the pitch-catch mode, the number of transducers used in the UDM system is halved. 

**Figure 2:**
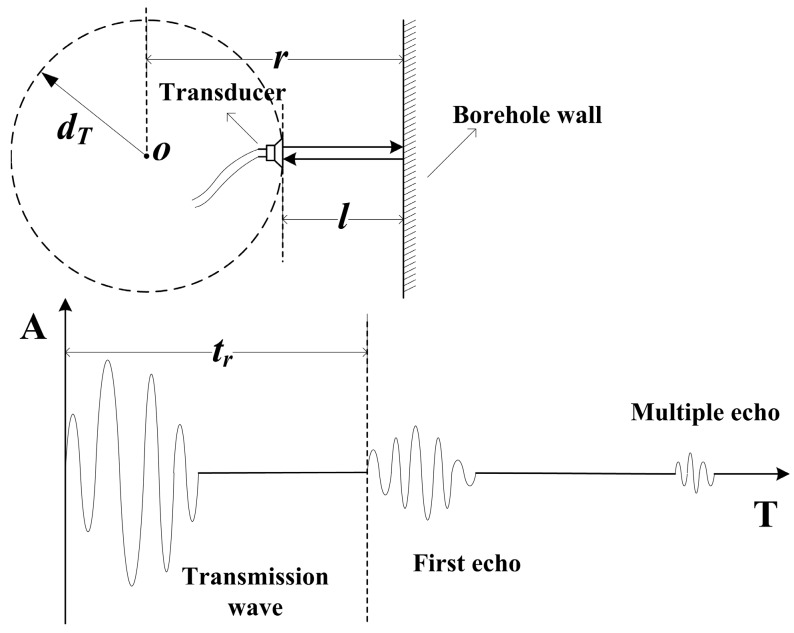
Principle diagram of the pulse-echo mode ultrasonic distance measurement system. *O* is the tool axis, *d_T_* is the distance between the tool axis and the transducer, *l* is distance between the transducer and the borehole wall, and *t_r_* is the arrival time of the first echo.

As shown in Figure 2, an ultrasonic transducer transmits ultrasonic waves to the borehole wall when excited by voltage pulses. The waves propagate through the mud medium (acoustic velocity is *ν_m_*) and reflected by the borehole wall. Then, the reflected waves (echoes) are received by the transducer.

The shortest distance between the tool axis and the borehole wall can be calculated based on the arrival time of the first echo, as follows:

(1) $${r\;=\;d}_{T}{\;+\;l\;=\;d}_{T}\;+\;\frac{1}{2}\nu_{m}t_{r}$$

In the case of a constant *ν_m_*, the length of *r* depends on the arrival time of the first echo.

**Figure 3:**
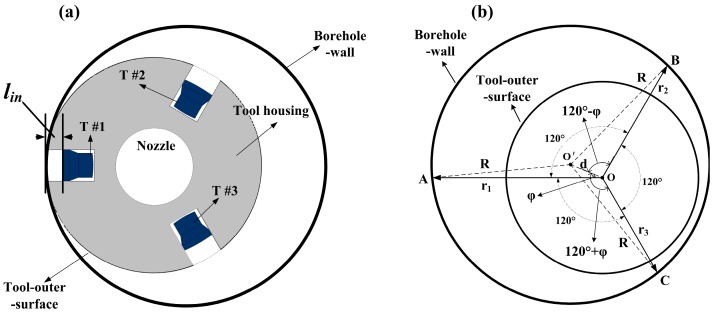
Profile map of the ultrasonic LWD caliper. (**a**) Transducer distribution; and (**b**) equivalent geometric relation graph of the transducer distribution.

The caliper consists of three independent distance measurement systems based on the aforementioned principle. In Figure 3a, the transducers are indented in the tool housing to avoid the influence of the blind zone. In Figure 3b, ultrasonic waves transmitted by transducers T_1_, T_2_, and T_3_ are reflected by the borehole wall, and the nearest reflection points are *A*, *B*, and *C*, respectively. Reflecting interface distances *l*_1_, *l*_2_, and *l*_3_ can be measured when the three distance measurement systems are working concurrently. According to Equation (1), the distances between the tool axis and each nearest reflection point can be obtained as follows: $r_{1}{\;=\;d}_{T}{\;+\;l}_{1}$, $r_{2}{\;=\;d}_{T}{\;+\;l}_{2}$, and $r_{3}{\;=\;d}_{T}{\;+\;l}_{3}$. The circumcircle of points *A*, *B*, and *C* is uniquely determined, and its radius and center are also derived. Let symbols *O′* and *R* denote the borehole axis and borehole radius, respectively; tool eccentricity $\bar{O\prime O}\;=\;d$; and eccentric angle ∠O′OA = φ. On the basis of the cosine theorem, relationships are observed among triangles Δ O′OA, Δ O′O B and Δ O′O C [7]:

(2) $$\{\begin{array}{l} R^{2}=d^{2}+{r_{1}}^{2}-2dr_{1}\cos\varphi \\ R^{2}=d^{2}+{r_{2}}^{2}-2dr_{2}\cos\left(120-\varphi \right)\\ R^{2}=d^{2}+{r_{3}}^{2}-2dr_{3}\cos\left(120+\varphi \right) \end{array}$$

To facilitate calculation and representation, the temporary variable $T=R^{2}-d^{2}$ is defined. Then, the borehole radius, tool eccentricity, and eccentric angle can be obtained by solving Equation (2):

(3) $$\{\begin{array}{l} T=R^{2}-d^{2}=\frac{r_{1}r_{2}r_{3}(r_{1}+r_{2}+r_{3})}{r_{1}r_{2}+r_{2}r_{3}+r_{1}r_{3}}\\ \varphi ={cos}^{-1}(\frac{{r_{1}}^{2}-T}{2r_{1}d})\\ d^{2}=\frac{{\left[r_{3}({r_{2}}^{2}-T)-r_{2}({r_{3}}^{2}-T)\right]}^{2}}{12{r_{2}}^{2}{r_{3}}^{2}}+\frac{{\left({r_{1}}^{2}-T\right)}^{2}}{4{r_{1}}^{2}}\\ R^{2}=d^{2}+T \end{array}$$

When correlating Equation (3) with Equation (1), the borehole radius and tool eccentric position at the current logging depth can be evaluated by measuring the arrival time of the echoes.

## 3. Acoustic Emission Technique for Reducing Blind Zone

### 3.1. Generation Mechanism and Theorical Calculation of the Blind Zone in a UDM System

As mentioned earlier, three piezoelectric ceramic transducers are adopted in the tool. During the excitation stage, an electric pulse with a certain frequency is applied to the transducer, which generates a mechanical vibration that causes the transducer to emit ultrasonic waves. When the excitation pulse is removed, the transducer continues to vibrate due to mechanical inertia. Such a vibration is called the residual vibration or “tailing” of the transducer. During the tailing stage, the echo signal is superimposed on the residual vibration of the transducer, which cannot be distinguished from the residual vibration until the vibration stops or is sufficiently small. Then, half of the acoustic travel distance is called the blind zone length of the UDM system.

If tailing time is *t_b_*, then blind zone size *l_b_* can be calculated using:

(4) $${l\;}_{b}=\frac{1}{2}{\;\nu \;t}_{b}$$

where *ν* is the acoustic velocity of the medium. 

**Figure 4:**
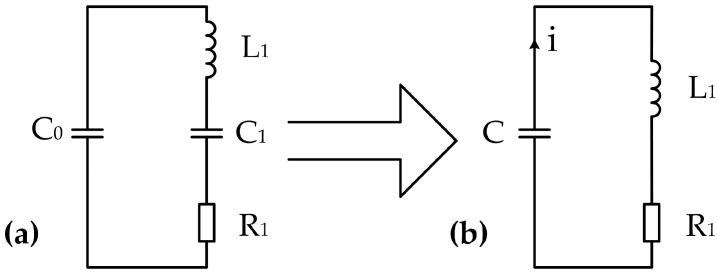
Equivalent circuits of the transducer: (**a**) general form; and (**b**) series-resonant form.

The residual vibration of transducers is a type of underdamping vibration, and the blind zone can be reduced if the vibration is rapidly attenuated. The ultrasonic transducer has an equivalent circuit, as shown in Figure 4a. The entire circuit can be regarded as an *RLC* underdamping oscillation model, where *R*_1_ is the dynamic resistance, also known as the mechanical loss resistance, and *C*_0_, *C*_1_, and *L*_1_ are the static capacitance, dynamic capacitance, and dynamic inductance, respectively. If the transducer is excited by an electric pulse with amplitude *U*_0_ and frequency $f_{s}=1/2\pi \sqrt{L_{1}C_{1}}$, then series-resonance occurs in the branch *L*_1_*C*_1_*R*_1_. This branch has minimum impedance and functions as a pure resistance, where *f_s_* is the series-resonant frequency of the transducer. When the excitation pulse is removed, *C*_0_ and *C*_1_ are connected in series. Let ${C=C}_{0}C_{1}{/(C}_{0}{+C}_{1})$, then the equivalent circuit can be transformed into the form shown in Figure 4b. Capacitor *C* is used as the energy storage element and is discharged through *R*_1_ and *L*_1_. Voltage *u_c_* on capacitor *C* is a function of time, and its initial value is *U*_0_, which means $u_{c}\left(0\right){=U}_{0}$.

In the circuit shown in Figure 4b, the voltage at *C* is equal to the sum of the voltages at *R*_1_ and *L*_1_; that is:

(5) $$u_{c}{=u}_{l}{\;+\;u}_{r}$$

There are relationships $u_{l}{=™L}_{1}{di/dt=™L}_{1}{Cd}^{2}u_{c}{/dt}^{2}$ and $u_{r}{=™R}_{1}{i=™R}_{1}{\mathrm{Cdu}}_{c}{/d}_{t}$, then Equation (5) can be rewritten as:

(6) $$L_{1}C\frac{d^{2}u_{c}}{{\mathrm{dt}}^{2}}{\;+\;R}_{1}C\frac{{\mathrm{du}}_{c}}{\mathrm{dt}}{\;+\;u}_{c}=0$$

Voltage at *C* at a certain time can be evaluated by solving the second-order differential equation:

(7) $$u_{c}(t)=\frac{U_{0}}{\omega_{d}\sqrt{L_{1}C}}e^{{-\alpha }_{d}t}{\sin(\omega }_{d}t\;+\;\varphi )$$

**Figure 5:**
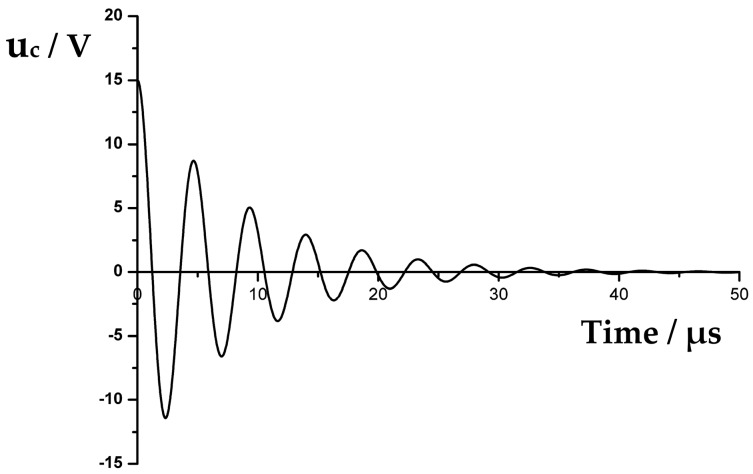
Residual vibration waveform of the transducer.

In Equation (7), $\alpha_{d}=R_{1}/2L_{1}$, $\omega_{s}=2\pi f_{s}$, $\omega_{d}=2\pi f_{d}=\sqrt{{\omega_{s}}^{2}{{-\alpha }_{d}}^{2}}$, and $\varphi =\tan^{-1}(\omega_{d}/\alpha_{d})$. As shown in Equation (7), *u_c_* decays exponentially along with time, and simultaneously, voltage oscillates periodically with angular frequency *ω_d_*. Table 1 displays the main impedance–admittance parameters of a given transducer, where *Q* and *K_eff_* are the quality factor and effective electromechanical coupling coefficient, respectively. As shown in Figure 5, the residual vibration waveform of the transducer can be drawn by using the aforementioned parameters.

Let variable *τ* represent the time when *u_c_* decays from its maximum amplitude $\frac{U_{0}}{\omega_{d}\sqrt{L_{1}C}}$ to a value that 10^−5^ times of the amplitude. Then:

(8) $$\frac{U_{0}}{\omega_{d}\sqrt{L_{1}C}}e^{{-\alpha }_{d}\tau }{=10}^{-5}\frac{U_{0}}{\omega_{d}\sqrt{L_{1}C}}$$

Variable *τ* can be obtained by solving Equation (8), as follows:

(9) $$\tau =\frac{{\ln10}^{-5}}{\alpha_{d}}\;\approx \;23\frac{Q}{\omega_{d}}$$

where $Q=\omega_{d}L_{1}/R_{1}$. When the parameters in Table 1 are inputted to Equation (9), we can obtain τ ≈ 98 μs. Then, the blind zone length *l_b_* can be calculated by combining this result with that of Equation (4). If the acoustic velocity of the medium is 1600 m/s, then $l_{b}=0{.5\;\nu }_{m}\tau \;\approx \;78.5\;\mathrm{mm}$.

A large blind zone is harmful to measuring borehole diameter, and residual vibration must be reduced as quickly as possible. To solve this problem, an acoustic emission circuit is provided.

### 3.2. Circuit Design for Reducing Blind Zone 

In general, the underdamping oscillation frequency of a transducer is similar to its series-resonant frequency. In accordance with the parameters in Table 1, the calculated frequency *f_d_* is 214 kHz, which is extremely close to *f_s_* (221 kHz). Hence, the residual vibration can hardly be distinguished from echo signal via filtering. Equations (7) and (9) show that the methods for increasing attenuation coefficient *α_d_* and reducing excitation pulse voltage *U_0_* can reduce the residual vibration. However, *α_d_* is determined by the impedance characteristics of the transducer, which cannot be changed by the external circuit. The reduction of emission power will reduce the detection range of the UDM system. Therefore, these two methods are undesirable. 

**Figure 6:**
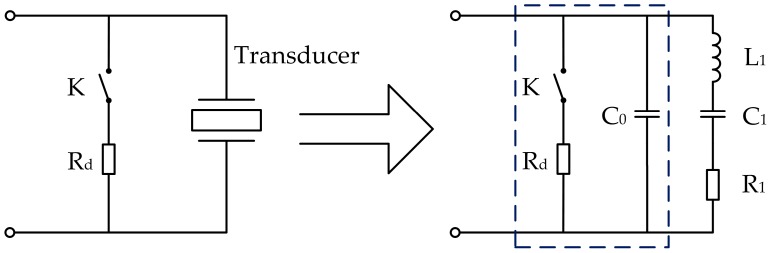
Model of the residual vibration reduction and its equivalent circuit.

The energy stored in the static capacitor *C*_0_ must be released quickly to reduce residual vibration. As shown in Figure 6, the solution adopted for this circuit design is as follows: connecting the transducer in parallel to a switch *K* and a resistance *R_d_* in series. During the excitation stage, switch *K* is off and *R_d_* cannot access the loop. When the excitation pulse is removed, switch *K* is closed, *R_d_* and *C*_0_ form an *RC* discharge circuit, and energy stored in *C*_0_ is consumed by *R_d_*. The time constant of the *RC* discharge circuit is *τ_d_*, and $\tau_{d}=R_{d}C_{0}$. Moreover, when *τ_d_* is smaller, the discharge time is shorter. Evidently, to eliminate residual vibration as soon as possible, the smallest *R_d_* should be selected while ensuring that the current flowing through *K* is lower than the maximum pulse current that can be tolerated by the switch.

**Figure 7:**
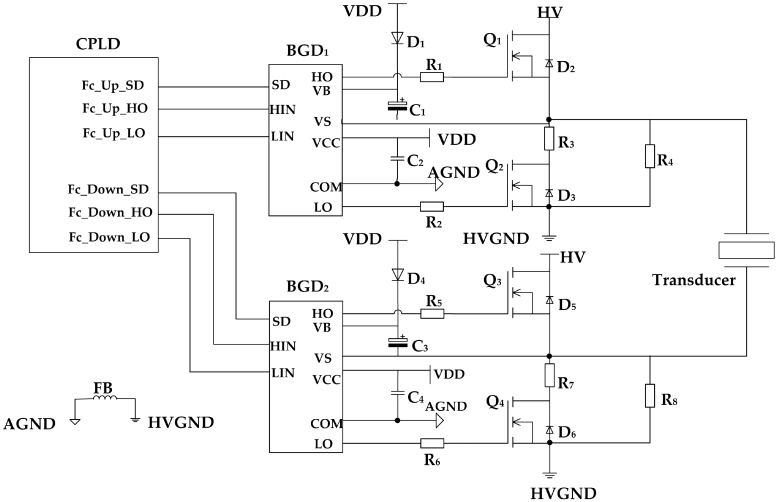
Schematic diagram of the acoustic emission circuit.

The role of switch *K* can be replaced by a high-speed N-channel MOSFET (NMOS). As shown in Figure 7, NMOSs *Q*_1_, *Q*_2_, *Q*_3_, and *Q*_4_ form a full bridge excitation circuit. Freewheeling diodes *D*_2_, *D*_3_, *D*_5_, and *D*_6_ are connected to the source and drain of NMOSs, which will protect the NMOSs from the reverse current produced by their sudden shutdown. According to the command of the complex programmable logic device (CPLD), bootstrap gate drivers *BGD*_1_ and *BGD*_2_ control the on-off function of the NMOS by adjusting *V_gs_* (NMOS gate–source voltage) [8,9].

**Figure 8:**
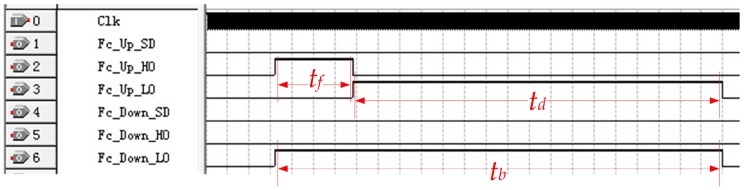
Control sequence of CPLD. *t_f_* is the excitation pulse width and $t_{f}=1/2f_{s}$; *t_d_* is the discharge duration of the transducer; The blind zone duration $t_{b}=t_{f}+t_{d}$, during which echo signal detection is not performed.

**Figure 9:**
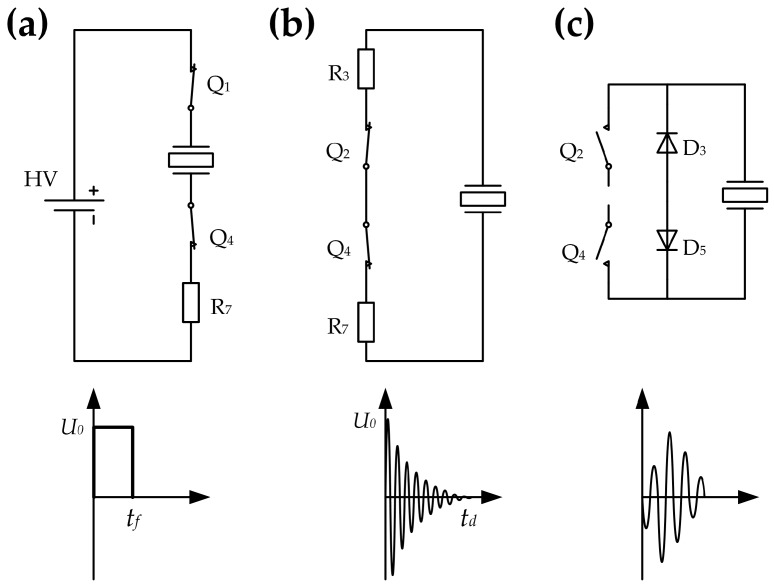
Simplified diagrams of the transducer excitation circuit at different stages: (**a**) acoustic emission stage; (**b**) discharge stage; and (**c**) echo signal detection stage.

The control sequence of the CPLD is shown in Figure 8. During the acoustic emission stage *t_f_*, the CPLD controls the bootstrap drivers *BGD*_1_ and *BGD*_2_ to turn on *Q*_1_ and *Q*_4_, and turn off *Q*_2_ and *Q*_3_. The voltage of pin *V_s_* of the bootstrap driver is pulled to *HV* and *HVGND* levels, respectively; and the transducer is excited by a rectangular pulse with amplitude *HV* and frequency *f_s_*. A simplified diagram of the excitation circuit at this stage is provided in Figure 9a. After excitation, *Q*_1_ and *Q*_3_ are turned off, whereas *Q_2_* and *Q_4_* are turned on. The circuit is simplified into the form shown in Figure 9b, and the transducer is discharged through resistors *R*_3_ and *R*_7_. The residual vibration of the transducer has been eliminated outside *t_b_*. During this stage, all NMOSs are turned off to prepare for echo detection, and the simplified circuit is shown in Figure 9c.

### 3.3. Circuit Test

**Figure 10:**
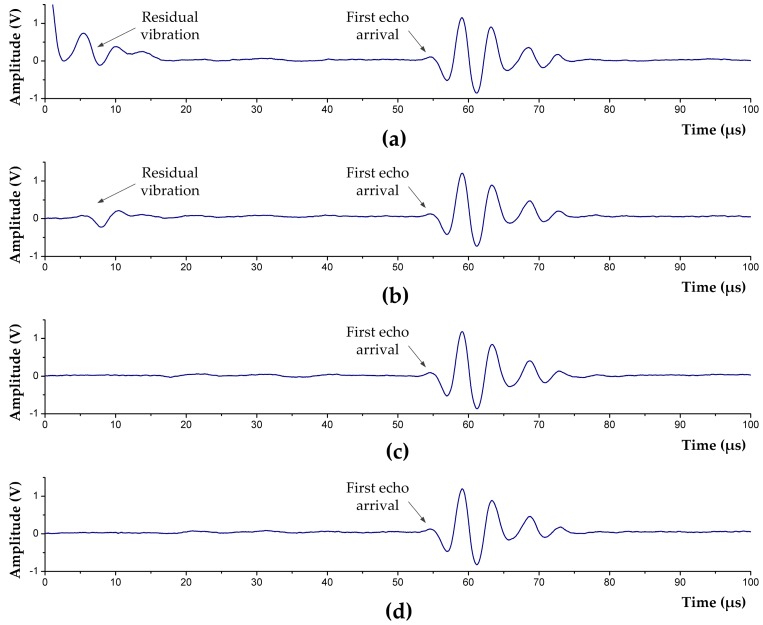
Test results of the acoustic emission circuit at different discharge duration *t_d_*. (**a**) *t_d_* = 0 µs; (**b**) *t_d_* = 5 µs; (**c**) *t_d_* = 10 µs; and (**d**) *t_d_* = 12 µs.

To test the function of the emission circuit and the actual blind zone length, a reasonable CPLD control sequence is designed; thus, the circuit can produce a rectangular single pulse (15 V, 2.25 μs) to excite the transducer. Figure 10 shows the waveforms after the excitation pulse being removed. Observed by a digital comparator, when *t_d_* is continuously changed, residual vibration is eliminated at $t_{d}\geq 10\;\mu s$, and it will not affect the echo detection anymore. 

In mud medium with acoustic velocity $\nu_{m}=1600\;m/s$, and blind zone time $t_{b}=t_{f}+t_{d}=12.25\;\mu s$, on the basis of Equation (4), the blind zone length can be calculated: $l_{b}=0.5\times 1600\;m/s\times 12.25\;\mu s=9.8\;\mathrm{mm}$, which shows that the blind zone of the UDM system has been significantly reduced.

During drilling and well logging, the influence of the blind zone on diameter measurement is inevitable when the outer surface of the tool is close to the borehole wall. As mentioned in Section 2 and Figure 3a, the transducers are indented into the tool housing to cope with this situation. “Zero distance” measurement can be realized as long as the indentation length $l_{\mathrm{in}}>9.8$ mm. When the mechanical structure of indented-in transducers is adopted, the equivalent length of the blind zone can be considered zero.

## 4. Echo Signal Compensation Technique

As mentioned in Section 1, the signal dynamic range is wide in diameter loggings, which may produce amplifier saturation overload, thereby causing the amplifier output to exceed the input range of the A/D converter. If an amplification circuit is present [10,11,12,13], whose gain changes with *l*, i.e., the distance between the sound source and the reflecting interface, then the dynamic range of the amplified signal can be reduced, and the diameter alteration effect on measurement is diminished. Evidently, this amplification circuit is highly beneficial for detecting echo signals.

### 4.1. Principle of Time-Varying Amplification

The main factors that cause acoustic attenuation are geometric diffusion, medium absorption, and scattering loss. The directivity of a high-frequency circular piston transducer is strong, and the acoustic waves propagating along the normal direction of the transducer plane can be regarded approximately as plane waves [14,15], i.e., geometric diffusion can be ignored, and acoustic attenuation is mainly determined by medium absorption and scattering loss. In this case, the amplitude of acoustic waves decrease exponentially with increasing propagation distance [16]. From the acoustic emission to the arrival of the first echo, the total travel distance of acoustic waves is 2*l*, and the signal amplitude of the first echo is as follows:

(10) $${U=R}_{e}{\mathrm{EU}}_{0}e^{-2\alpha l}$$

where *Re* is the reflection coefficient of the interface; *E* is the electromechanical coupling coefficient of the transducer; *U_0_* is the excitation pulse voltage; and *α* is the acoustic attenuation coefficient of the mud medium, which is determined by the mud density and acoustic wave frequency. 

It can be concluded from Equation (10) that if the mud density, acoustic wave frequency, and reflecting interface material are known, then the echo signal amplitude is determined only by distance *l*. When Equation (10) is combined with Equation (1):

(11) $${U=R}_{e}{\mathrm{EU}}_{0}e^{{-\alpha \nu }_{m}t}$$

where *α* and *ν_m_* are the parameters that are related only to the property of mud. Let ${\beta =\alpha \nu }_{m}$, then *β* is a constant in the specific mud medium. The echo signal amplitude *U* can be regarded as a function of travel time *t*, and Equation (11) can be rewritten as:

(12) $${U(t)=R}_{e}{\mathrm{EU}}_{0}e^{-\beta t}$$

From Equation (12), in the situation of constant mud property, *U* is related only to travel time *t*. Based on this relationship, a time-varying amplification method is proposed for detecting echo signals, which indicates that the gain of the signal amplification circuit can be changed regularly according to arrival time of echo, and the amplitudes of the output signal compensated by the circuit are consistent. Let *C_u_(t)* be the voltage compensation of the echo signal at time *t*. Then:

(13) $${U(t}_{1}{)C}_{u}{(t}_{1}{)=U(t}_{2}{)C}_{u}{(t}_{2}{)=\cdot \cdot \cdot =U(t}_{n}{)C}_{u}{(t}_{n})$$

Let *C* be the voltage compensation constant of the echo signal at the initial time of echo detection. Then:

(14) $${U(0)C}_{u}{(0)=U(t)C}_{u}(t)$$

when Equations (12) and (14) are merged:

(15) $$R_{e}{\mathrm{EU}}_{0}\times C=R_{e}{\mathrm{EU}}_{0}e^{-\beta t}\times C_{u}\left(t\right)\Rightarrow C_{u}\left(t\right)=e^{\beta t}C$$

*K* and *B* are set as constants, where K=20lnβ, and B=20logC, then further derivation provides the gain compensation at time *t* as follows:

(16) $$C_{g}\left(t\right)=20\log C_{u}\left(t\right)=K\;t\;+\;B$$

Equation (16) shows that the gain compensation curve of the echo signal is a straight line with a certain slope (constant *K*). Time-varying compensation can be realized by linearly adjusting the gain of the amplification circuit as the law in Equation (16). The amplitude of the compensated signal is only determined by the excitation voltage and the reflection coefficient of the interface, and not related to travel time.

### 4.2. Circuit Design for Time-Varying Amplification

**Figure 11:**
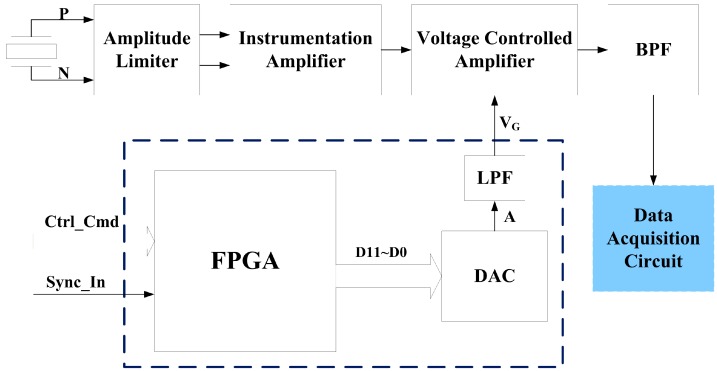
Block diagram of the time-varying amplification circuit. Ctrl_Cmd and Sync_In are the control command and acquisition synchronization signals sent from the main control circuit, respectively.

Figure 11 presents the block diagram of the time-varying amplification circuit. The core parts of the circuit include the voltage controlled amplification (VCA) circuit and the gain control voltage (V_G_) generation circuit. The VCA circuit amplifies input signal according to V_G_. The V_G_ generation circuit generates a V_G_ curve that varies with echo arrival time based on the principle of time-varying amplification.

The gain control voltage generation circuit mainly consists of a field-programmable gate array (FPGA) and a digital-to-analog converter (DAC). A number of gain compensation curves are stored in the on-chip ROM of the FPGA to match the mud medium with different densities. When detecting the acquisition synchronization signal, the FPGA chooses a corresponding compensation curve according to the received control command [17]. The 12-bit digital values of compensation voltage are read out from the ROM and transmitted to the DAC, where the digital values are converted into analog voltages [18]. After filtering via a low-pass filter (LPF), the gain control voltage *V_G_* is finally generated.

The amplitude limiter consists of clamping diodes and resistive dividers, high-voltage emission pulses and large echo signals are limited within a reasonable range by the amplitude limiter to protect subsequent circuits. The instrumentation amplifier converts a differential signal into a single-ended signal. Then, the single-ended signal is amplified by the voltage controlled amplifier, and the gain of this amplifier is linearly changed along with *V_G_*:

(17) $$\mathrm{Gain}(\mathrm{dB})={\mathrm{kV}}_{G}+b$$

when Equations (17) and (16) are combined, *V_G_* at time *t* can be derived as follows:

(18) $$V_{G}=\frac{K}{k}t+\frac{B-b}{k}$$

Ambient noise and amplifier noise coupled with amplified signals will interfere with echo detection. Hence, a band-pass filter (BPF, 150–300 kHz) is adopted to suppress these noises. After amplifying and filtering, the analog signal is finally outputted to the data acquisition circuit for discretization, processing, and storage.

All of the devices used in the circuit should be able to adapt to the working environment; that is, they should have a small package, high temperature tolerance, and high pressure tolerance. Moreover, the power supply for LWD tools originates from batteries when it is working downhole. Hence, electronic devices with low power consumption are preferred to improve the continuous working time of the tool.

### 4.3. Circuit Test

**Figure 12:**
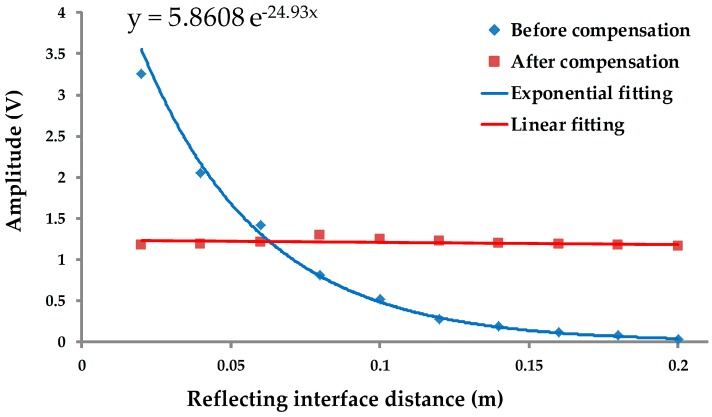
Comparison chart of the signal amplitudes (peak-to-peak) before and after compensation.

To verify the practical results of the echo signal compensation circuit, a comparison test was carried out in a water-based mud medium (1520 m/s, 1.25 g/mL). Figure 12 presents the signal amplitudes of the first echoes that are reflected by the interfaces, and the interface distances range from 20 mm to 200 mm. By exponential fitting, an attenuation curve of echoes before compensation was drawn. After compensation, the amplitudes are basically consistent and are rarely related to the propagation distance or arrival time.

According with the maximum amplitude and the minimum amplitude of echo signals, the dynamic ranges of echo signals before and after compensation can be calculated to be 40.7 dB and 1.2 dB, respectively. The dynamic range of echo signals is reduced by 39.5 dB.

**Figure 13:**
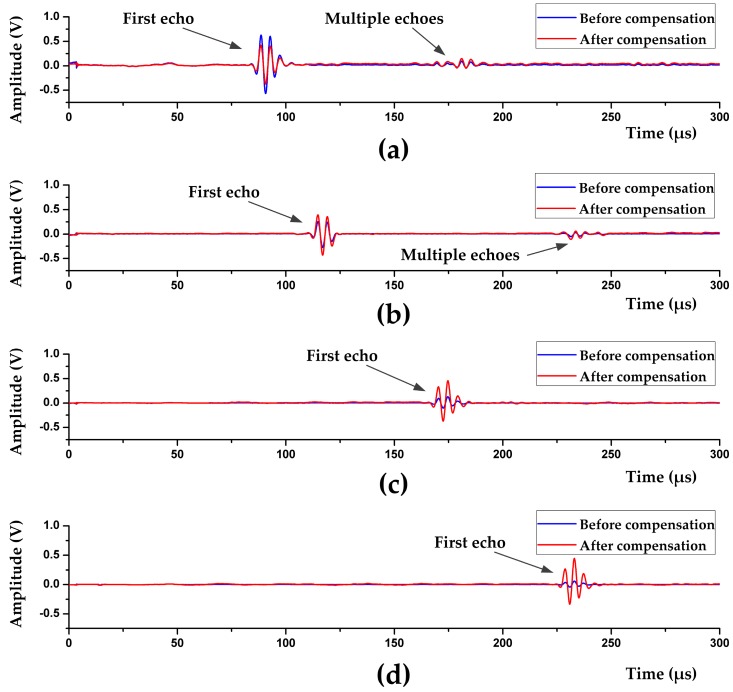
Waveforms of the echo signals reflected by interfaces at different distance: (**a**) 60 mm; (**b**) 80 mm; (**c**) 120 mm; and (**d**) 160 mm.

As shown in Figure 13, the first echoes and multiple echoes are all compensated by the circuit. The amplitudes of the first echoes are consistent, and the amplitudes of multiple echoes are smaller due to the multiple reflection losses and coupling losses.

## 5. Conclusions

The performance of an ultrasonic LWD caliper depends highly on the functions of its electronic system. Research on the electronic system also provides important guidance for designing the mechanical structure. To realize a “zero distance” measurement, a mechanical structure with indented-in transducers is designed, which solves the measurement problem caused by borehole wall contact. When the blind zone is smaller, the indentation length is shorter, and the influence on the mechanical strength of the drill collar is less. Therefore, in addition to choosing high-performance transducers, blind zone length was reduced by optimizing the design of the acoustic emission circuit and enhancing the capability of the caliper to detect near the reflecting interface. As mentioned in Section 1 and Section 3.3, the blind zone length in [7] and this design are 25 mm and 9.8 mm, respectively. The comparison shows that the blind zone length is reduced by 60%.

In the case of borehole wall collapse or tool eccentricity, diameter alteration will cause a change in the echo signal amplitude, and ordinary program-controlled amplification circuits cannot cope with this situation. However, the gain of time-varying amplification can be changed along with the echo arrival time to compensate for the acoustic attenuation. Therefore, the influence of the propagation distance is eliminated, and the signal dynamic range of the amplified echo is significantly reduced, which is very helpful in signal detection and the processing of subsequent circuits.

## Figures and Tables

**Table 1:** Main impedance–admittance parameters of a given transducer.

*f_s_*/kHz	*Q*	*K_eff_*	*R*_1_/Ω	*C*_0_/nF	*C*_1_/nF	*L*_1_/mH
221	5.25	0.47	974	0.75	0.16	4.16
